# Investigation of the hydrodynamic properties of a new MRI-resistant programmable hydrocephalus shunt

**DOI:** 10.1186/1743-8454-5-8

**Published:** 2008-04-21

**Authors:** David M Allin, Marek Czosnyka, Hugh K Richards, John D Pickard, Zofia H Czosnyka

**Affiliations:** 1 Shunt Evaluation Laboratory & Academic Neurosurgical Unit, Addenbrooke's Hospital, P.O. Box 167, Hills Road, Cambridge CB2 2QQ, UK

## Abstract

**Background:**

The Polaris valve is a newly released hydrocephalus shunt that is designed to drain cerebrospinal fluid (CSF) from the brain ventricles or lumbar CSF space. The aim of this study was to bench test the properties of the Polaris shunt, independently of the manufacturer.

**Methods:**

The Polaris Valve is a ball-on-spring valve, which can be adjusted magnetically *in vivo*. A special mechanism is incorporated to prevent accidental re-adjustment by an external magnetic field. The performance and hydrodynamic properties of the valve were evaluated in the UK Shunt Evaluation Laboratory, Cambridge, UK.

**Results:**

The three shunts tested showed good mechanical durability over the 3-month period of testing, and a stable hydrodynamic performance over 45 days. The pressure-flow performance curves, operating, opening and closing pressures were stable. The drainage rate of the shunt increased when a negative outlet pressure (siphoning) was applied. The hydrodynamic parameters fell within the limits specified by the manufacturer and changed according to the five programmed performance levels. Hydrodynamic resistance was dependant on operating pressure, changing from low values of 1.6 mmHg/ml/min at the lowest level to 11.2 mmHg/ml/min at the highest performance level. External programming proved to be easy and reliable. Even very strong magnetic fields (3 Tesla) were not able to change the programming of the valve. However, distortion of magnetic resonance images was present.

**Conclusion:**

The Polaris Valve is a reliable, adjustable valve. Unlike other adjustable valves (except the Miethke ProGAV valve), the Polaris cannot be accidentally re-adjusted by an external magnetic field.

## Background

New models of hydrocephalus shunts are continuously being released onto the health-care market [1-5]. Yet these new designs do not always match the needs of the patient suffering from hydrocephalus. For example: many valves have very low hydrodynamic resistance but, without siphon-preventing mechanisms, they cause over-drainage [2]. Also, some shunts may present with reflux at low flow [3]. In some valves adjustable settings can be changed accidentally [5,6]. One recently raised criticism was that many of the magnetically-adjusted shunts in use can be altered by an external magnetic field. This includes weak sources created by home appliances [6,7], as well as the more obvious, stronger fields encountered during magnetic resonance (MR) scanning [8-10]. The Polaris valve is an adjustable hydrocephalus shunt, which incorporates a mechanism that allegedly prevents accidental re-adjustment in a magnetic field of up to 3 Tesla.

The aim of this study was to measure the properties of the shunt, in order to provide neurosurgeons with independent, reliable and accurate data about its performance. The long-term stability of a valve's behaviour was tested in a laboratory environment that mimics, at least in part, conditions within the human body. The tests are able to demonstrate whether the shunt is susceptible to alterations in CSF drainage caused by postural changes, by external magnetic fields, by changes in ambient temperature, or by the presence of a pulsating pattern in the inlet pressure.

Where possible, we have stated whether specific shunt properties revealed during the tests may be considered useful or detrimental to the restoration of the CSF circulation. Such an assessment may encourage or discourage the use of the shunt in the management of specific types of hydrocephalus.

## Methods

### Shunt description

The Polaris Programmable Valve (Sophysa Ltd, Orsay, France) is a differential-pressure valve, the opening pressure of which can be magnetically adjusted after implantation using a magnetic tool supplied with the valve.

**Figure 1:**
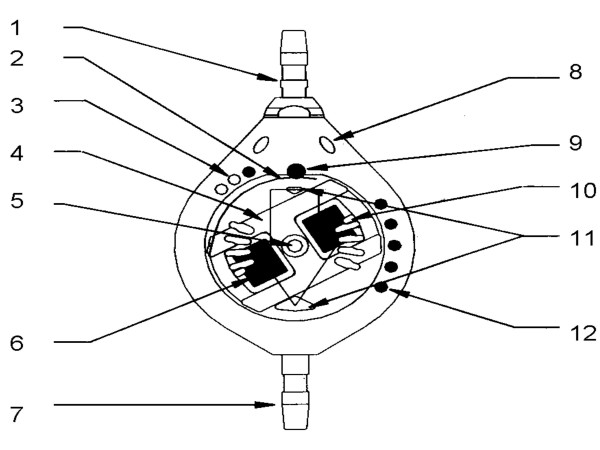
**Schematic diagram of construction of Sophysa Polaris Valve (Figure scanned from the leaflet provided by the manufacturer).** 1: inlet connector, 2: semicircular spring; 3 & 12: radiopaque setting identification points, 4: rotor, 5: ruby axis, 6: micromagnet, 7: outlet, 8: fixation holes, 9: ruby ball, 10: adjustment lugs, 11: safety stop.

CSF flows through the inlet which is closed by a ruby ball (Figure 1: 9) sitting in a cone, and supported by a flat semi-circular spring (Figure 1: 2). When the inlet pressure increases, the ruby ball rises out of the cone and CSF flows into the rigid main fluid container and then into the distal catheter.

The tension of the flat spring can be adjusted non-invasively by moving the rotor with the external programming magnet. The rotor is composed of two permanent magnets that are not susceptible to demagnetisation. The rotor shifts the end of the spring between the consecutive indexing notches, thereby changing the working pressure. The pre-load or performance level is claimed, by the manufacturer, to increase the performance pressure in a linear fashion from 30, 70, 110, 150 to 200 mmH_2_O. Manufacturers traditionally use mmH_2_O as units to express valve pressure settings, whereas in the most hydrocephalus-related publications, pressure is expressed in mmHg. The pressure settings of the Polaris valve correspond to 2.3, 5.2, 8.1,11.1 and 14.7 mmHg.

In comparison to the older Sophysa programmable valve, which was susceptible to accidental re-programming by an external magnetic field (stronger than 40 mT), the Polaris valve is equipped with a patented self-locking magnetic system. It contains two mobile micromagnet shuttles, which attract each other when in the resting position. In this position the rotor is blocked by the lugs sliding in between adjustment notches. An adjustment instrument containing a magnet with a uniquely profiled magnetic field is required to move the rotor. When the adjustment tool is in the proper position the shuttles are pushed outward, unblocking rotor, which can then be moved to change the performance levels (Figure 1).

### Testing rig

**Figure 2:**
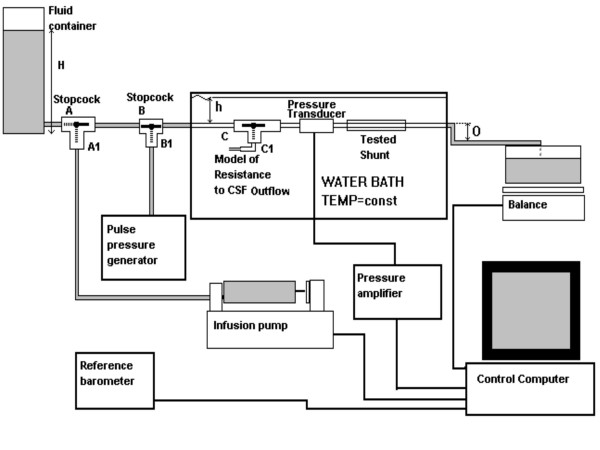
A diagram of the test rig showing the main components.

The hydrodynamic properties of the shunts are described by various parameters such as opening pressure, closing pressure, resistance to fluid flow, pressure-flow performance, etc. Our testing methods and definitions of the hydrodynamic parameters have been described in previous articles and reports [11], but for better understanding the description is repeated below (Figure 2). The shunts under test were submerged in a water bath at a constant temperature at a defined depth (h). The working fluid, deionised and de-aerated water, was supplied by the fluid container or the infusion pump. A pulse pressure of controlled amplitude created by the pulse pressure generator could be added to the static pressure. The viscosity and specific gravity of water reflect the physical properties of CSF under normal conditions.

In hydrocephalus, the resistance to CSF outflow in the patient is usually increased and finite. A model of resistance to CSF outflow could be added to the circuit before the shunt, to study the shunt's performance in conditions mimicking the *in vivo *environment (called 'residual resistance'). Pressure before the shunt was measured with an absolute pressure transducer (Gaeltec Luer Lock transducer, Gaeltec Ltd, Scotland). The fluid flowing through the shunt was collected in a container placed on the electronic balance. Measurement of flow was recorded on a standard IBM compatible personal computer that read and zeroed the balance to calculate the flow rate every 15s. By this method the weight of the outflowing fluid was measured incrementally, which negated any effect of fluid vaporisation from the outlet container, since vaporisation over 15s period was considered to be negligible. The computer also analysed the pressure waveform from the pressure transducer and controlled the rate of the infusion pump. The effect of changes in atmospheric pressure was compensated for by using the reference barometer, such that the effective pressure was measured as current pressure minus drift of atmospheric pressure.

The shunt and pressure transducer were placed at the same height. The water column in the fluid container (H), the degree of the shunt submersion (h) and the level of the outlet tubing (O) could be changed according to the test protocol.

### Test protocol

**Figure 3:**
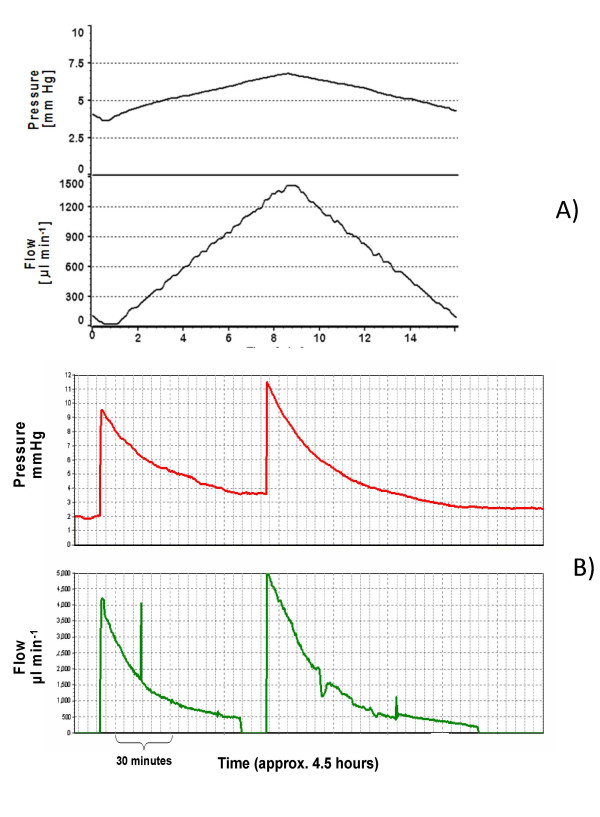
**Typical plots of flow and pressure over time: **A: Flow-pressure test. Flow is the controlled variable and pressure is the measured variable. B: Pressure-flow test. Pressure is the controlled variable and flow the measured variable.

The shunt was tested under two different regimes:

*(i) *The differential pressure was measured while flow through the shunt was varied (flow-pressure, Figure 3A).

*(ii) *The flow through the shunt was measured while the differential pressure across the shunt was varied (pressure-flow, Figure 3B).

Three Sophysa Polaris shunts (set at 70 mmH_2_O, equivalent to 5.2 mmHg) were filled with deionised and de-aerated water. Air bubbles were gently flushed out, according to the manufacturer's instructions. The shunts were then mounted in three identical rigs (Figure 2). Before each test, the shunts were inspected for air bubbles and gently flushed if necessary, and the pressure transducers and reference barometers zeroed.

The following parameters were calculated:

• Closing pressure: The differential pressure below which flow through the shunt ceases. The closing pressure was measured as the intercept of the regression line with the x-axis, drawn between pressure (independent variable) and flow (dependent variable) for flow from 0.2 to 0.05 ml/min.

• Opening pressure: The differential pressure above which non-zero flow through the shunt was measured during the ascending ramp of the infusion pump rates.

• Hydrodynamic resistance: The change in pressure divided by the change in flow decreasing from about 1.2 to 0.3 ml/min. The resistance was measured as a linear regression gradient between pressure and flow. This parameter describes the resistance of the permanently opened shunt.

• Operating pressure at 0.3 ml/min flow: The pressure measured during infusion of fluid at 0.3 ml/min, which is approximately equivalent to CSF flow.

The above parameters were measured at all the different adjustable pressure levels (10 tests at each setting for each valve)

Additionally parameters were compared at three temperatures (30, 36 and 40°C, three repeated tests). Pulse waveform given from the generator of graded peak-to-peak amplitude (1 to 50 mmHg, at 60 cycles per minute) was used to measure influence of proximal pulsations on operating pressure. Negative outlet pressure of -19 mmHg was applied to mimic siphoning in upright position.

### MRI analysis

The magnitude of magnetic field translational attraction was assessed using the standardized procedure of the so-called deflection angle test. The valve was suspended by a piece of lightweight thread that was attached to a plastic protractor so that the angle of deflection from the vertical of a line could be measured. Assuming the mass of the thread to be negligible, a quantitative estimate of the translational attraction of the valve was calculated.

Artefacts were determined using a spherical gel-filled phantom. The T1 and T2 values for this gel were similar to those of grey matter. MRI was performed using a 3 Tesla MR system (MAGNETOM Tim Trio, SIEMENS, Erlangen, Germany) and a 12-channel head matrix radio frequency receiver coil and whole body transmit coil. The following pulse sequences were used: 1. T1 weighted spin echo, TR/TE 500/20 ms, matrix size 256 × 256, 4 mm slice thickness, 22 cm FOV, 2 excitations; and 2. Gradient echo pulse sequence, TR/TE 500/20 ms, flip angle 20, matrix size 256 × 256, 4 mm slice thickness, 22 cm FOV, 2 excitations. Artefact volume was expressed in cm^3^. All image display parameters were carefully selected to facilitate a valid determination of the artefact size.

### Statistical methods

Mean values, standard deviations and maximal-minimal values were used to express average parameters and their spread for the three shunts. To evaluate fluctuations of parameters in altered conditions a paired t-test for parameters at a baseline and in altered conditions was used. The t-test was used to evaluate sample-related differences in parameters.

Analysis of variance (ANOVA), with time as the independent factor, was used to evaluate the stability of parameters over time. The level *p *< 0.05 was used as the limit for statistical significance.

## Results

### Valve under normal conditions with opening pressure set at performance level 70 mmH_2_O (5.2 mmHg)

The mean value of the shunt opening and closing pressures (these two pressures were identical) was 6.7 mmHg with a range of 5.0 to 7.1 mmHg (tests repeated 30 times in 3 valves). The addition of a distal catheter did not change the opening and closing pressure significantly (10 tests in 3 valves).

**Figure 4:**
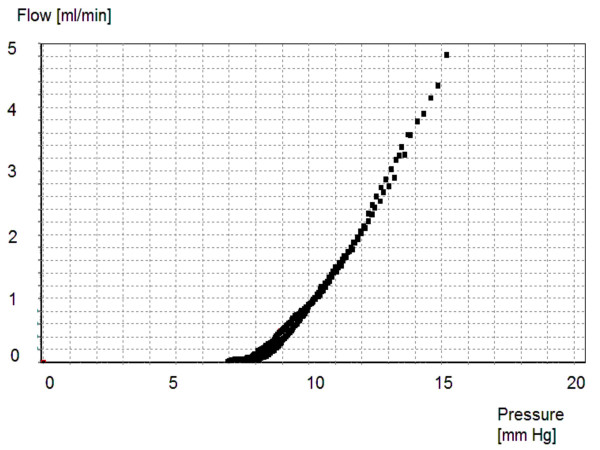
Individual pressure-flow curves for the Polaris Valve without a distal catheter and set at 70 mmH_2_O (5.2 mmHg).

**Figure 5:**
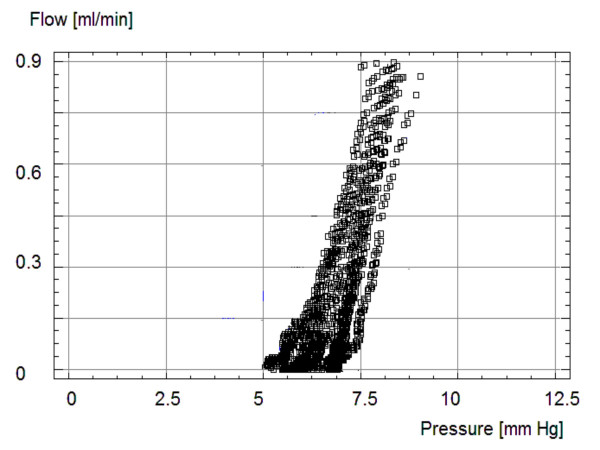
Superimposed pressure-flow curves taken from 10 tests utilizing one shunt at 70 mmH_2_O (5.2 mmHg). The scatter of the measurement points indicates good agreement over repeated measurements.

A typical pressure-flow curve with pressure plotted along the x-axis and flow along the y-axis, is shown in Figure 4. The valve without a distal catheter, and with no pulsatile pressure wave, had slightly non-linear characteristics. The hydrodynamic static resistance was equivalent to the inverse of the gradient. Composite pressure-flow curves (10 tests in 3 valves) from one valve are presented in Figure 5 as flow versus pressure.

The hydrodynamic resistance was 2.06 ± 0.41 mmHg/ml/min (30 tests in 3 valves). This is a low value, and is approximately 2–3 times lower than physiological resistance to CSF outflow. The resistance increased to 5.12 ± 0.76 mmHg/ml/min after the connection of the distal catheter (110 cm long; ID 1.1 mm).

Operating pressure was stable and consistent; the mean value was 6.7 mmHg, range from 6.1 to 8.5 mmHg (30 tests in 3 valves).

**Figure 6:**
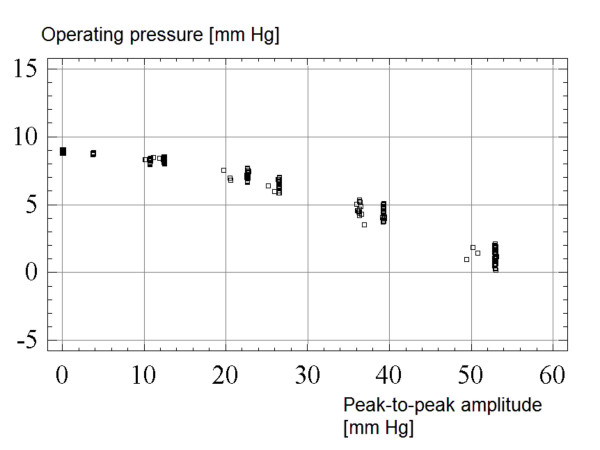
**The effect of pulse amplitude: A plot of the relationship between operating pressure (y-axis) and peak-to-peak pulse amplitude (x-axis).** The test was repeated 3 times in 3 valves.

A pulse pressure with variable peak-to-peak amplitude of 1 to 50 mmHg produced a significant decrease in operating pressure of around 3 mmHg (Figure 6; test repeated 3 times in 3 valves). It is predicted that flow through the valve after implantation may be affected by the pulsatile component of CSF pressure.

None of the parameters (opening, closing pressure and resistance) were altered by a temperature change from 30°C to 40°C. Therefore we would not expect a change in CSF drainage even during a high fever or when ambient temperature is low.

The Polaris Valve increases CSF drainage rate when a negative outflow pressure is applied. By decreasing outlet level by 20 cmH_2_O, flow increased by around 4 ml/min. None of the parameters was altered by changing the residual resistance to CSF outflow.

### Effect of programming

Programming of the valve was checked using both pressure-flow and flow-pressure tests. Good conformity between the pressure-flow curves and the nominal data was found.

**Figure 7:**
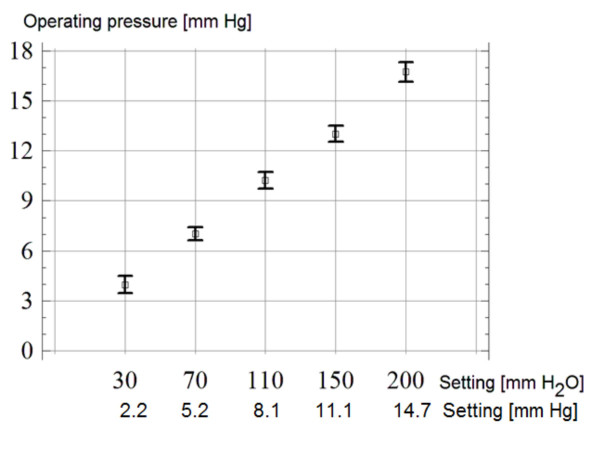
**Mean values and 95% confidence limits for the valve's closing pressure at different performance levels.** The test was repeated 10 times in 3 valves.

Operating pressures (for flow of 0.3 ml/min) and 95% confidence limits at different programming levels are shown in graphical form in Figure 7 (10 tests in 3 valves at each setting). Checking the position of the valve, settings and re-adjustments was easy and reliable. The hydrodynamic resistance depended on operating pressure and increased with higher settings. Nominal values are given in Table 1 for the shunt working without and with distal catheter.

**Table 1:** Hydrodynamic resistance of the opened shunt depends on the preset performance level.

Performance level	Shunt without Catheter	Shunt with catheter (110 cm long, internal diameter 1.1 mm)
30 mmH_2_O 2.3 mmHg	1.58 (0.3)	4.61 (.71)
70 mmH_2_O 5.2 mmHg	2.06 (0.41)	5.12 (0.76)
110 mmH_2_O 8.1 mmHg	2.48 (0.52)	5.62 (0.82)
150 mmH_2_O 11.1 mmHg	5.18 (0.81)	8.21 (1.01)
200 mmH_2_O 14.7 mmHg	11.2 (1.31)	14.2 (1.42)

Units are mmHg/ml/min; mean values and standard deviation (in parentheses). Each test was repeated 3 times in 3 valves.

### Influence of a magnetic field

**Figure 8:**
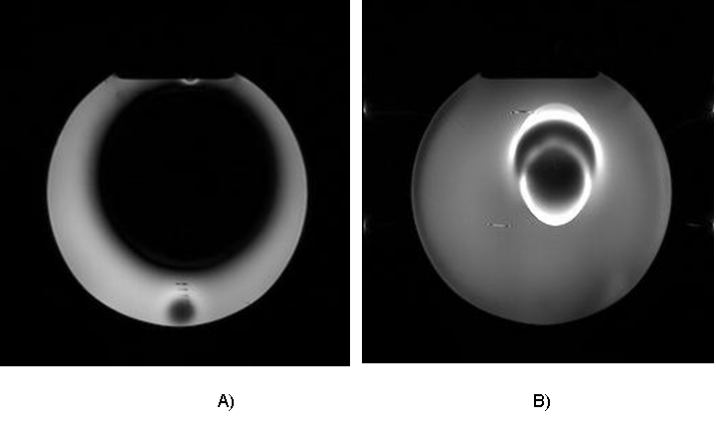
**Artifact on MR image: The Polaris valve was placed in a water-filled container, with diameter equivalent to adult skull).** A) Gradient echo image; B) T1 image.

The valve cannot be re-programmed by an external magnetic field of up to 3T (MRI magnet). It does not heat up when placed within the magnetic field. The maximal translational force measured was 81G. These values are still considered as safe after implantation. Distortion of the MRI scan was significant: gradient echo 954 cm^3^, and T1 100 cm^3 ^(Figure 8).

### Other valve properties

Opening and closing pressures displayed very limited variation during all the tests. The changes were not time-related.

When the valve was unpacked and filled for the first time with water it worked normally almost immediately, providing that all air bubbles had been removed.

The hydrodynamic resistance and operating pressure did not exhibit any time-related trends during the 30 days of testing. No significant (*p *> 0.05) differences in measured parameters were found between the three valves tested. All the values of closing pressures measured were within the limits specified by the manufacturer. The valve did not show any reflux when tested. It did not exhibit reversal of flow for an outlet-inlet differential pressure of up to 200 mmHg.

Assembled junctions (standard surgical sutures were applied by a trained neurosurgeon) did not break when a test specimen was subjected to a load of 1 kg force for 1 minute. All junctions remained free from leakage when the water pressure was increased to 3 kPa (about 25 mmHg).

## Discussion

The Polaris valve represents the next generation of the well-known Sophysa programmable valves. First released onto the market in 1985, these were the first valves in which the operating pressure could be adjusted transcutaneously. However, one design flaw that has consistently been a problem with programmable valves, has been their tendency to be reset by the external magnetic field created by domestic appliances [6,7] and by MRI scanners [8,10]. As there is only one other programmable valve that is able to resist magnetic resetting (Miethke ProGAV) [1,9], the Polaris will hopefully prove to be a welcome addition to the market. The translational forces observed in a 3T magnet appear to be safe for the patient. The artifact produced by the valve is, however, considerable. Implantation on the chest instead of the head might, therefore, be worth considering if the patient is to be scanned in the future.

The Polaris Valve is similar to the previously evaluated Sophysa Valve with regards to its hydrodynamic properties. The main difference being its aforementioned ability to resist MR induced resetting.

At the low opening pressure the valve has a low hydrodynamic resistance and as the valve does not have a siphon-control mechanism, it would be safer if the resistance was closer to a physiological value of 8–10 mmHg/ml/min. It might, therefore, be appropriate to use a thinner distal catheter when lower performance levels are required [12]. The hydrodynamic resistance increases with the pressure performance level. The resistance at 8.21 – 14.2 mmHg/ml/min matches and exceeds the normal CSF outflow resistance when the shunt is set at the highest pressure levels (150 to 200 mmH_2_O). This is consistent with manufacturer's recommendations to use a high pressure setting at the initial stage of the treatment, in order to minimize the risk of overdrainage and shunt dependency.

Even if the test demonstrates a limited decrease of the differential pressure with CSF pulse amplitude, it seems hazardous to speculate any clinical consequences without consideration of brain and distal compliance, and/or the type of ICP waveform.

As for any adjustable shunt, distortion of the MRI image is significant when the slice is crossing the Polaris valve. This should be taken into consideration if MRI examination is considered necessary for the follow-up after shunt implantation and should influence the location of the shunt placement accordingly. It should be remembered that the performance of the Polaris valve is not affected by the location of the implant on the skull. Regarding the suggestion to implant the Polaris valve on the chest rather than on the skull, the manufacturer is very cautious on this implantation site for the thickness of the skin covering the valve is likely to exceed 8 mm, making the unlocking of the valve impossible.

Laboratory testing of the valve provides knowledge of its hydrodynamic parameters. This data can be used for obtaining further functional information when testing the valve's performance *in vivo*, either by using an infusion test or with overnight CSF pressure monitoring [13,14]. Nevertheless, regardless of how well the valve performs in the laboratory, data should ideally be supplemented by clinical studies.

## Conclusion

The Polaris valve performed well in the *in vitro *testing protocols; the hydrodynamic properties were good and the valve pressure settings were MRI resistant. It presented a significant artifact in MRI scans. The valve should be closely monitored once used clinically, to ensure that its function corresponds to pre-clinical data obtained.

## Competing interests

This evaluation study was commissioned by Sophysa SA in, agreement with the University of Cambridge. The study was conducted independently and the presented results were not influenced by the manufacturer. MC is on unpaid leave from Warsaw University of Technology.

## Authors' contributions

DMA analysed the data and wrote the manuscript, ZHC and MC performed measurements, JDP and HKR supervised project and helped in data analysis and writing the manuscript. All authors have read and approved the final version of the manuscript.
